# Supplementary material for: Dansgaard-Oeschger cycles of the penultimate and last glacial period recorded in stalagmites from Türkiye
Source: Nat Commun. 2024 Feb 8;15:1183. doi: 10.1038/s41467-024-45507-5 (PMC10853552; doi:10.1038/s41467-024-45507-5)
Supplement: Supplementary file 1 — Supplementary Information [file 41467_2024_45507_MOESM1_ESM.docx]

Supplementary Information for

**Dansgaard-Oeschger cycles of the penultimate and last glacial period recorded in stalagmites from Türkiye**

F. Held, H. Cheng, R. L. Edwards, O. Tüysüz, K. Koç, D. Fleitmann

**Supplementary Fig. 1 Correlation coefficients of Sofular stalagmites.** Correlation coefficients calculated by the iscam algorithm^1^ for **a** δ^18^O and δ^13^C isotope profiles of stalagmites So-1 and So-2 covering the Holocene, **b** δ^18^O and δ^13^C isotope profiles of stalagmites So-1, So-2, So-4 and So-13 covering the last glacial period, and **c** δ^18^O and δ^13^C isotope profiles of stalagmites So-4 and So-57 covering the penultimate glacial period.


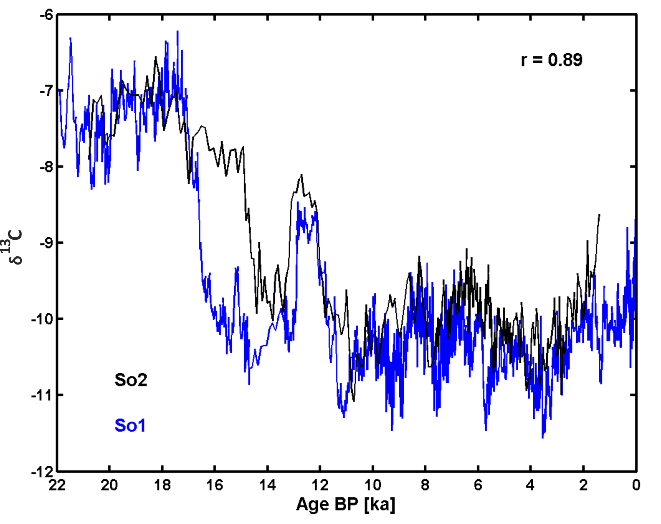

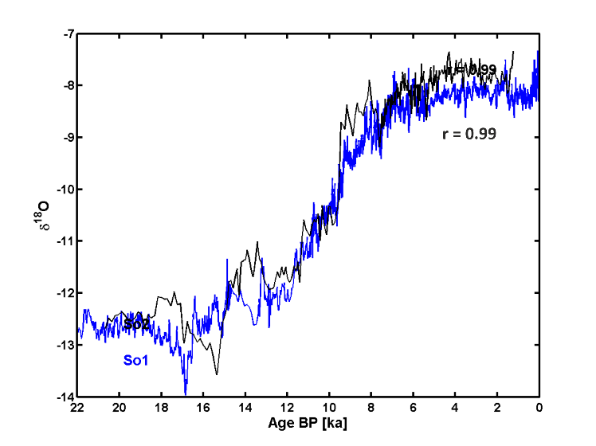

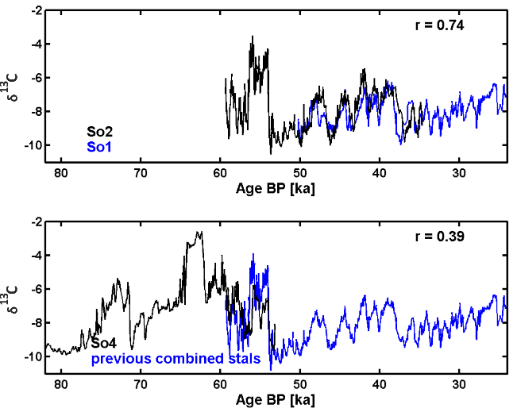

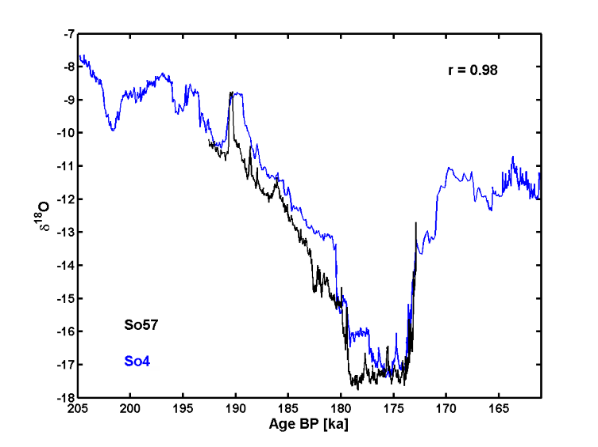

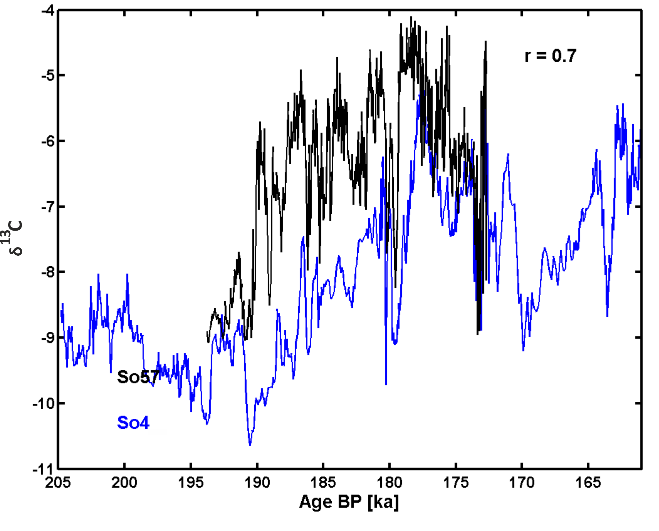

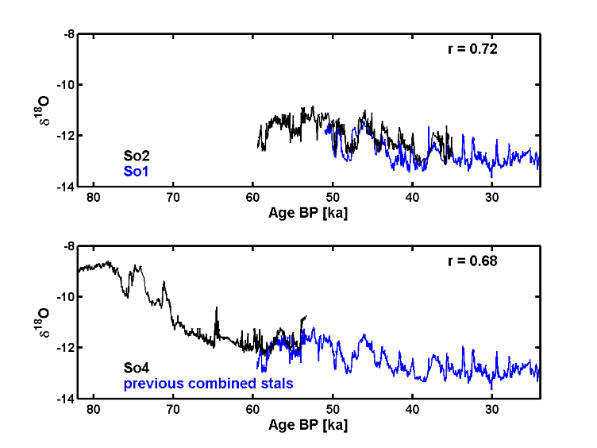

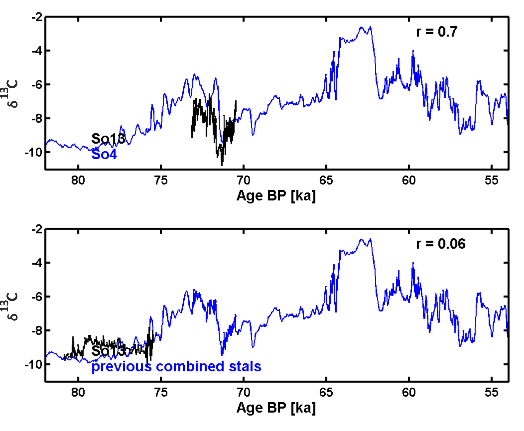

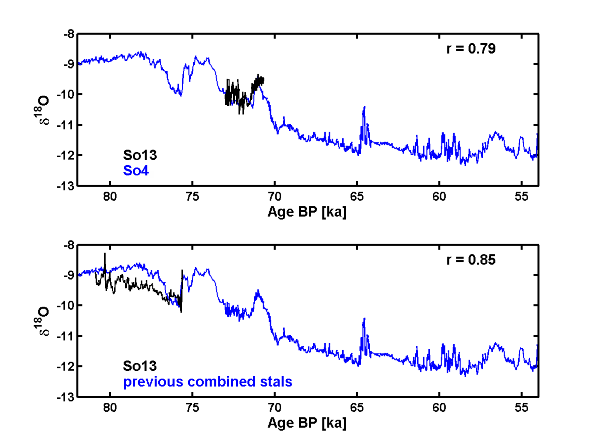


c)

b)

a)


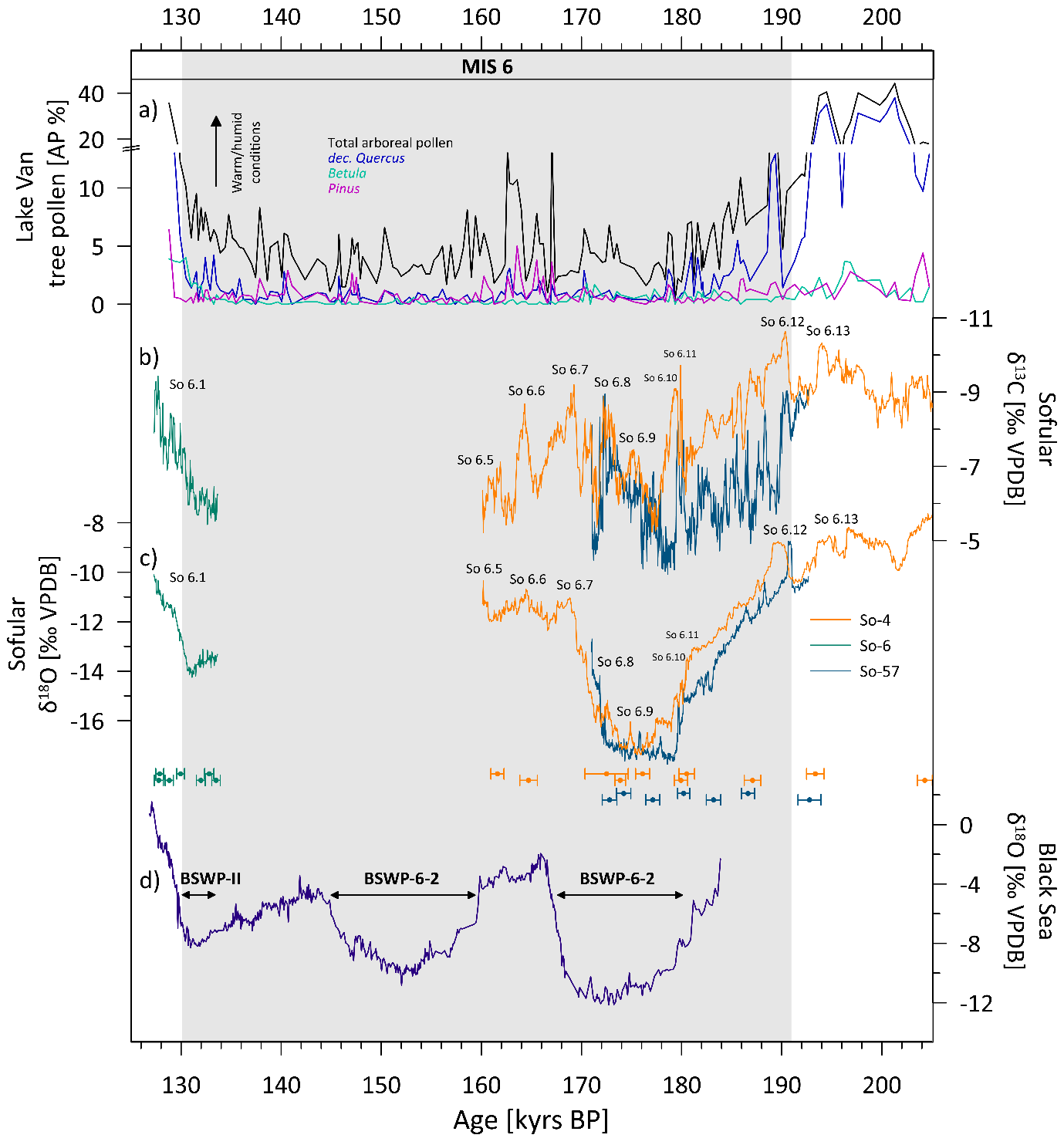


**Supplementary Fig. 2 Comparison between Sofular stable isotope profiles and regional proxy records. a** Arboreal pollen (AP) from Lake Van indicating environmental conditions^2^. Please note the break in the y-axis. **b** δ^13^C profiles of stalagmites So-4, So-6, So-57. Abrupt negative δ^13^C shifts associated with D-O events are consistent with vegetation oscillations (AP between ∼ 1 and 18 %) at Lake Van, indicating warmer and wetter interstadials and cooler and drier stadials. **c** δ^18^O profiles of stalagmites So-4, So-6, So-57. Colour coded ^230^Th ages with 2σ-error bars are plotted below. **d** δ^18^O_Ostracods_ profile from the Black Sea^3^. Black arrows denote meltwater periods. BSWP refers to Black Sea water pulses.


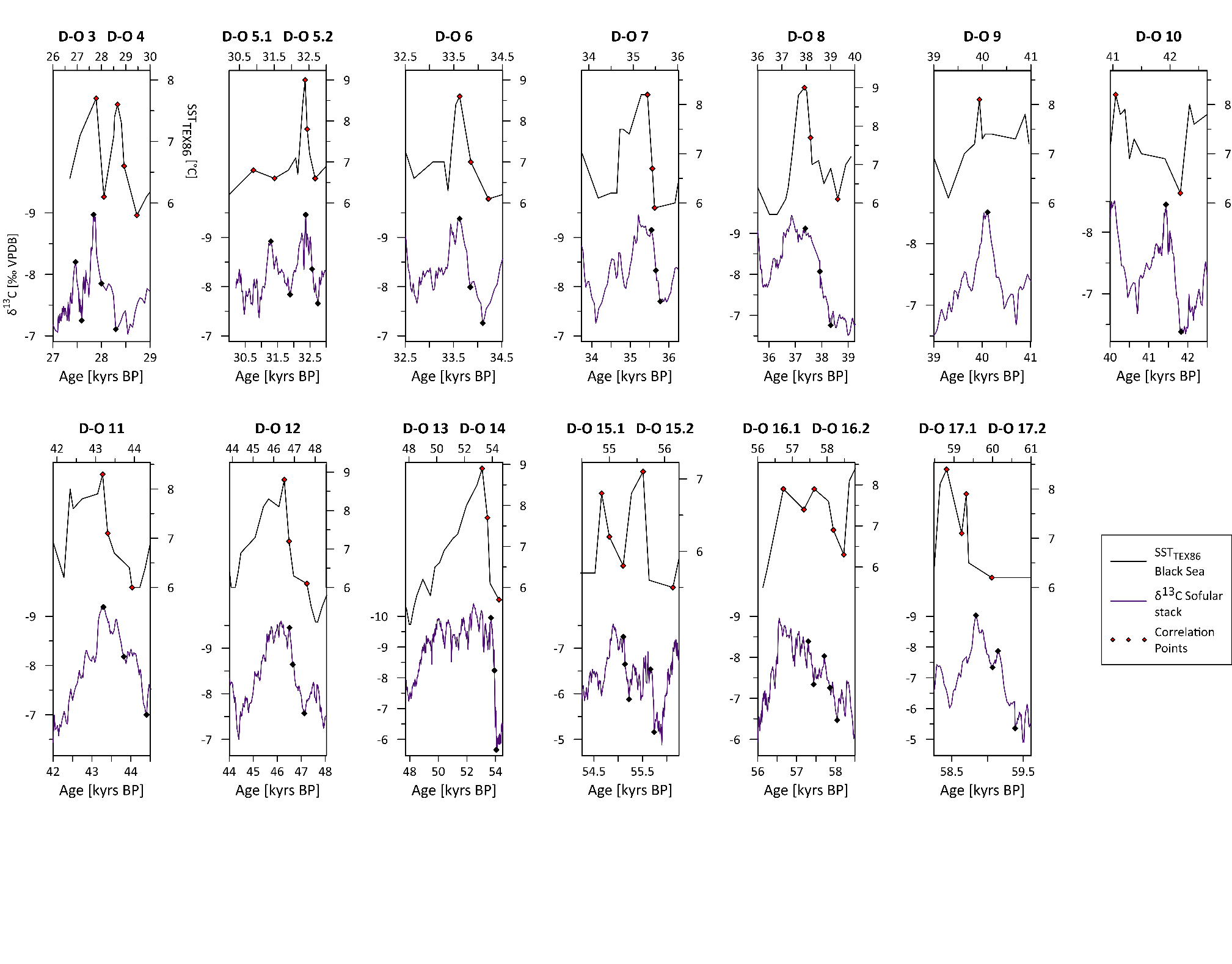


**Supplementary Fig. 3** **Data points used for the correlation of speleothem δ^13^C and sea surface temperatures (MIS 2-4).** Data points mark the onset, midpoint, and peak of D-O events in Sofular δ^13^C profiles and the SST record from the Black Sea during MIS 2-4.


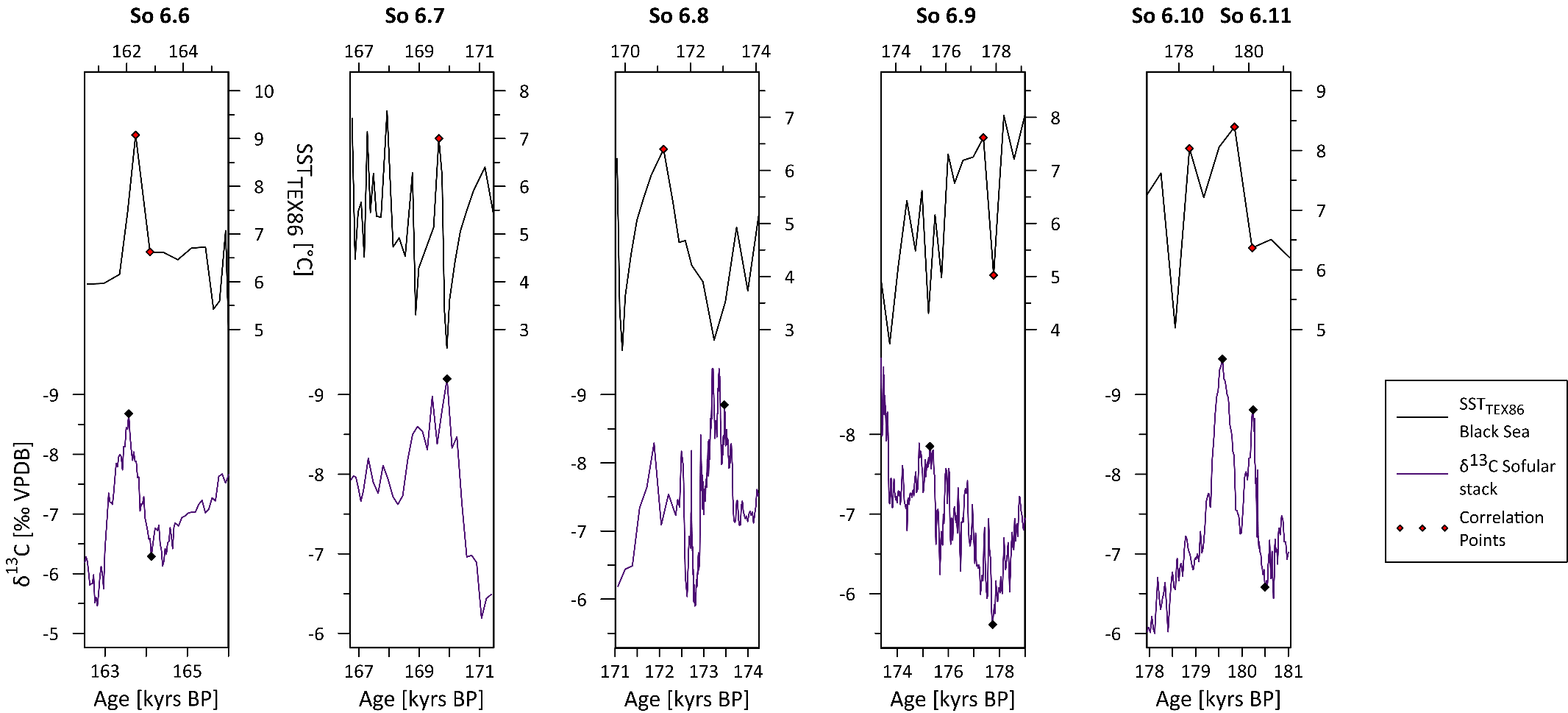


**Supplementary Fig. 4** **Data points used for the correlation of speleothem δ^13^C and sea surface temperatures (MIS 6).** Data points mark the onset and peak of D-O events in Sofular δ^13^C profiles and the SST record from the Black Sea during MIS 6.


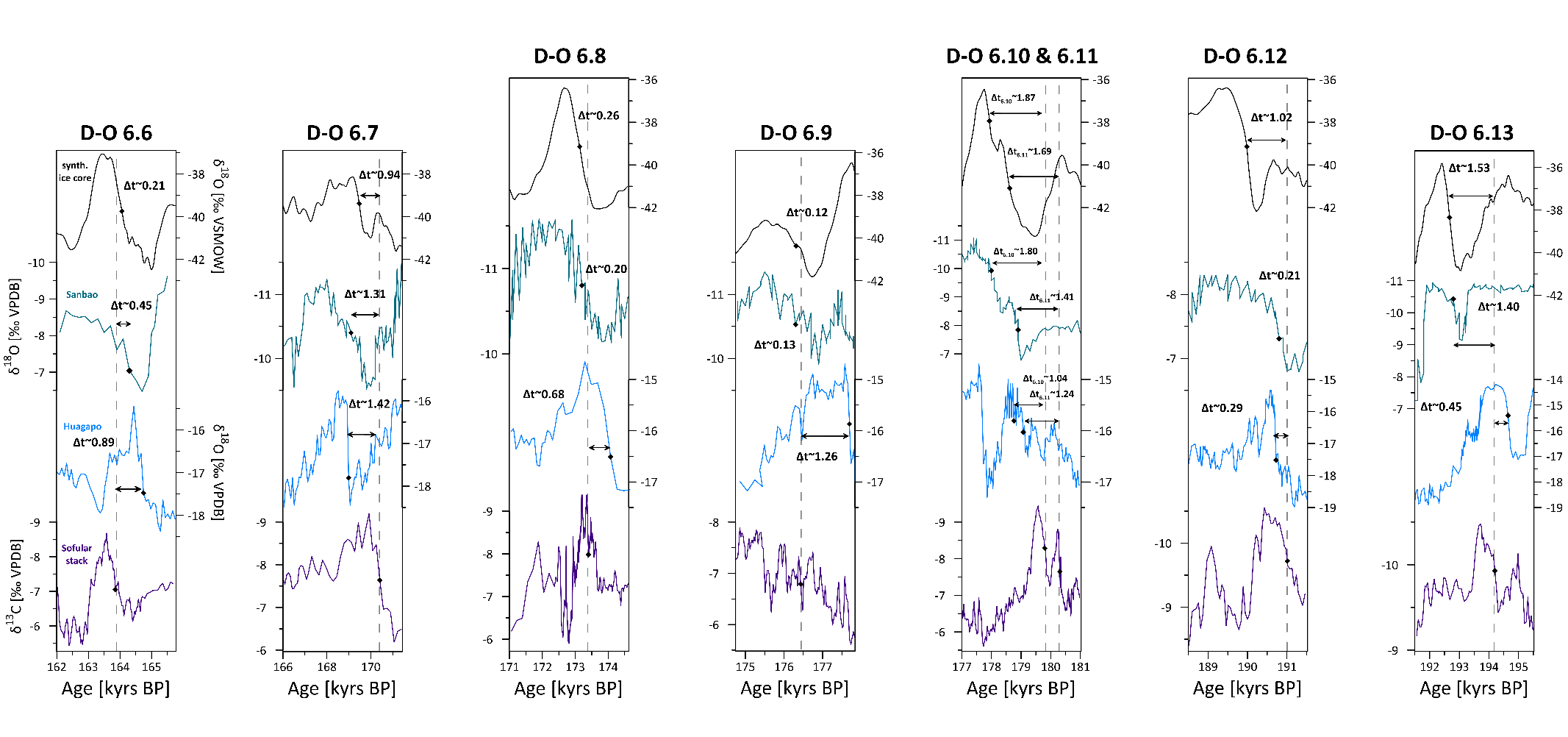


**Supplementary Fig. 5 Timing of D-O cycles during MIS 6.** The Sofular stack record (purple) was used as a reference to determine the age offset between the absolute dated speleothem records from Huagapo Cave in Peru*^5^* (blue) and Sanbao Cave in China*^6^* (green) as well as the synthetic ice core*^7^* (black). Data points indicate the midpoint of a D-O event at which the offset was determined.

**Supplementary Fig. 6 Data points used for calculating the pacing of D-O events during MIS 6.** **a** Synthetic ice core δ^18^O record^7^. **b** Sofular δ^13^C stacked record. **c** Huagapo speleothem δ^18^O record^5^. **d** Sanbao speleothem δ^18^Orecord^6^. Numbers above the records denote D-O events. Grey arrows and corresponding numbers denote the pacing of D-O events in kyrs. The pacing corresponds to the time between the midpoints (black dots) of two D-O events.


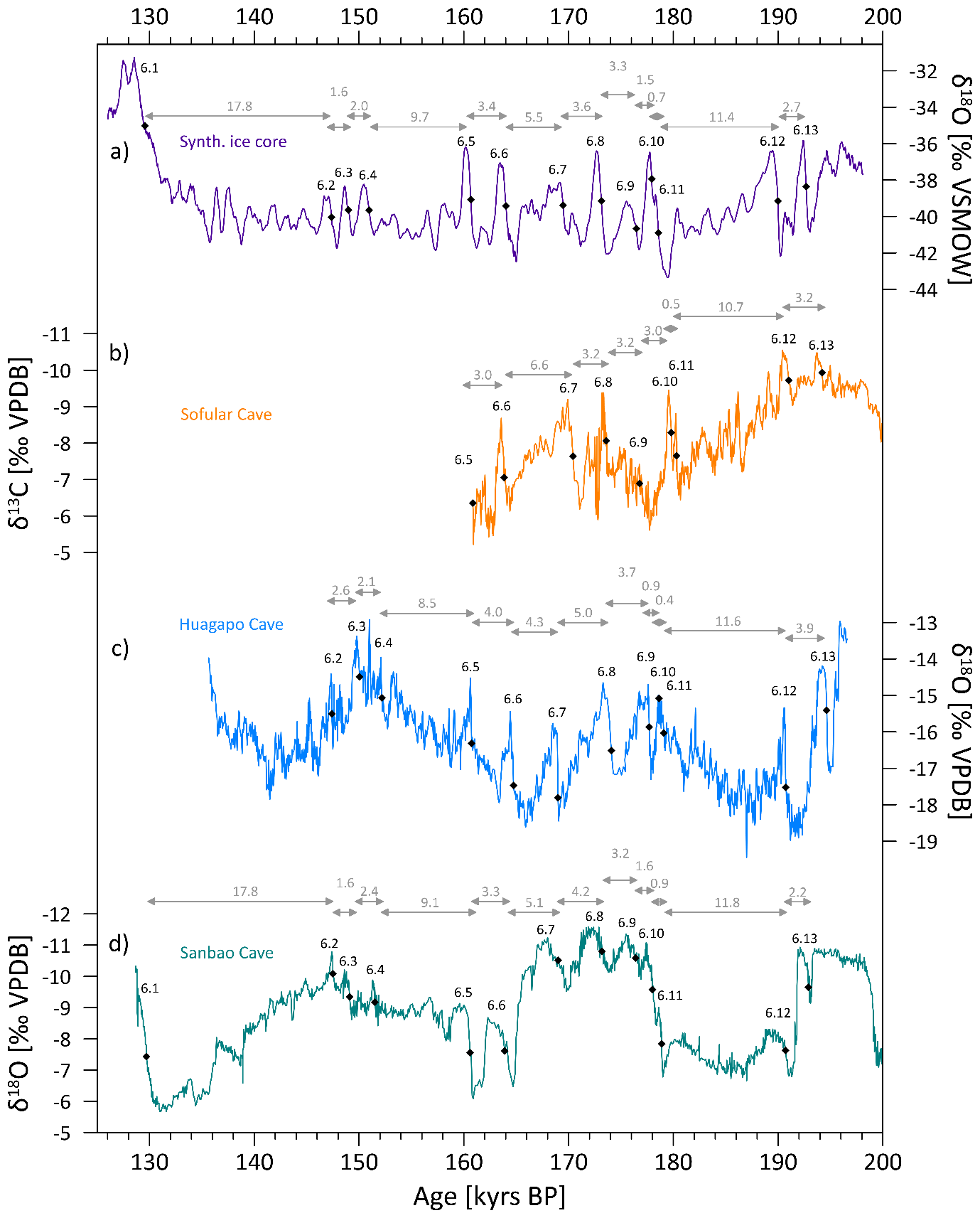


**Supplementary Fig. 7 Data points used for calculating the pacing of D-O events during MIS 2-4.** D-O pacing in the Sofular δ^13^C stacked record. Numbers above the records denote D-O events. Grey arrows and corresponding numbers denote the pacing of D-O events in kyrs. The pacing corresponds to the time between the midpoints (black dots) of two D-O events.


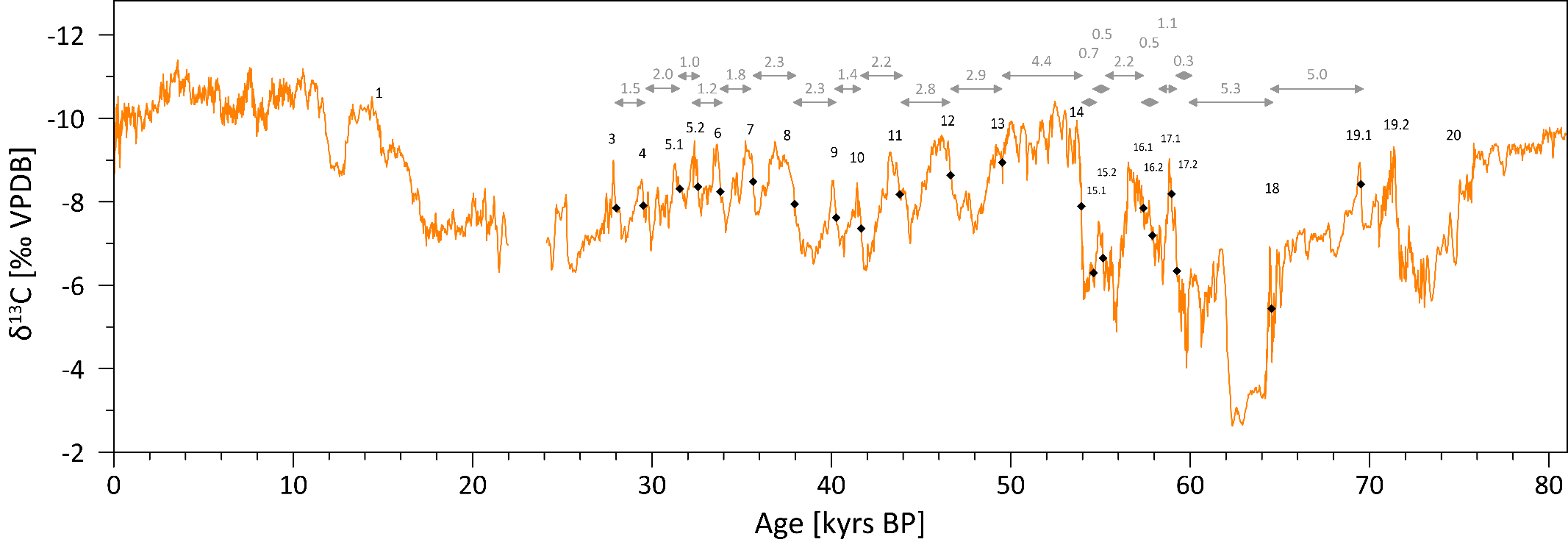


**Supplementary Text 1: Uranium-series dating at the Department of Geology and Geophysics, University of Minnesota and at the Xi'an Jiaotong University.** All ^230^Th dating of stalagmite So-1, So-2, So-4, So-6 and So-13 at the Minnesota Isotope Laboratory, University of Minnesota, USA, and stalagmite So-57 at the Xi'an Jiaotong University, China, was made on a multi-collector inductively coupled plasma mass spectrometers (MC-ICP-MS, Thermo-Finnigan-Neptune). The chemical procedures used to separate the uranium and thorium for ^230^Th dating are similar to those described in Edwards et al.^8^. Both uranium and thorium isotope analyses were performed on the multiplier behind the retarding potential quadrupole (RPQ) in peak jumping mode. The procedures for characterizing the multiplier were similar to those described in Cheng et al.^9^. Mass fractionations for thorium were determined by bracketing measurements of ^233^U - ^236^U in spike or sample solutions. The ^230^Th dating technique is described in detail in Cheng et al.^10^.

**Supplementary Text 2: Uranium-series dating at the Geological Institute, University of Bern.** For MC ICP-MS analyses, 0.1 - 0.2 g of powdered sample were spiked with a mixed ^229^Th-^236^U spike and dissolved in nitric acid and taken to dryness. U and Th were separated on anion columns using a 0.5 ml UTEVA resin bed. A first separation, using 7N nitric acid, produced a pure Th fraction and an impure U fraction, which was purified on the same columns using 5N hydrochloric acid. After evaporation, the fractions were placed in oxygen plasma for a minimum of 30 minutes to also remove organic residues from the plasma. U and Th mass spectrometry was done on a Nu Instruments multi-collector ICP-MS equipped with an ESI Apex (TR) desolvating system without membrane and using a self-aspirating element-specific nebuliser and disposable capillaries. With an uptake rate of ca 50 microliter/min the ion yield for U and Th was about 70V/ppm. U measurements were done from 0.5 N nitric acid solutions in static mode, whereby masses 236 and 234 were measured in parallel electron multipliers and 235 and 238 in Faraday cups. Baselines were taken on either side of the peaks and interpolated. The electron multiplier yield was calibrated every five samples by running a NIST U050 solution. The ^238^U/^235^U ratio was used for instrumental fractionation correction if the ^238^U signal was greater than 1 V; if smaller, the fractionation factor was input from bracketing standards. Normal washout time for U between samples was 5 minutes with 0.5 N nitric acid (< 1‰ memory); longer washout times were used where significant isotope differences between samples were expected. Runs on the NIST U960 standard yielded delta(^234^U/^238^U) -37.2 +/- 2.1‰ (1SD, N=35), where the equilibrium ratio is after Cheng et al.^9^. Th measurements were made from 3N hydrochloric solutions in a two-cycle multi-collector dynamic mode, whereby one electron multiplier, equipped with a WARP filter, alternately measured masses 229 and 230. U standard was added to Th run solutions for two reasons: first, to correction for instrumental mass fractionation, and second, to provide a reference isotope (238) to eliminate the effects of plasma flicker in obtaining the ^229^Th/^230^Th ratios. Variations of U and Th signals during the run are fully correlated if no organic matter is present. Baselines were measured at 229.5 and 230.5 for samples and standards with significant (>10exp-12A) ^232^Th. For stalagmites, the baseline was quite flat and measured at 230.5. Washout time was 15 min. to 1‰ of the Th signal if the capillary and nebulizer were free of organics.

**Supplementary References**

1. Fohlmeister, J. A statistical approach to construct composite climate records of dated archives. *Quat Geochronol* **14**, 48-56 (2012).

2. Pickarski, N. & Litt, T. A new high-resolution pollen sequence at Lake Van, Turkey: insights into penultimate interglacial–glacial climate change on vegetation history. *Climate of the Past* **13**, 689-710 (2017).

3. Wegwerth, A. et al*.* Major hydrological shifts in the Black Sea “Lake” in response to ice sheet collapses during MIS 6 (130–184 ka BP). *Quaternary Science Reviews* **219**, 126-144 (2019).

4. Bintanja, R. & van de Wal, R. S. W. North American ice-sheet dynamics and the onset of 100,000-year glacial cycles. *Nature* **454**, 869-872 (2008).

5. Burns, S. J., Welsh, L. K., Scroxton, N., Cheng, H., Edwards, R. L. Millennial and orbital scale variability of the South American Monsoon during the penultimate glacial period. *Sci Rep* **9**, 1234 (2019).

6. Cheng, H. et al. The Asian monsoon over the past 640,000 years and ice age terminations. *Nature* **534**, 640-646 (2016).

7. Barker, S. et al. 800,000 years of abrupt climate variability. *Science* **334**, 347-351 (2011).

8. Edwards, R. L., Chen, J. H., Wasserburg, G. J. U-238 U-234-Th-230-Th-232 Systematics and the Precise Measurement of Time over the Past 500000 Years. *Earth and Planetary Science Letters* **81**, 175-192 (1987).

9. Cheng, H., Edwards, R. L., Hoff, J., Gallup, C. D., Richards, D. A., Asmerom, Y. The half-lives of uranium-234 and thorium-230. *Chem Geol* **169**, 17-33 (2000).

10. Cheng, H. et al. Timing and structure of the 8.2 kyr BP event inferred from delta O-18 records of stalagmites from China, Oman, and Brazil. *Geology* **37**, 1007-1010 (2009).
